# The Relationship of Dehydration and Body Mass Index With the Occurrence of Atrial Fibrillation in Heart Failure Patients

**DOI:** 10.3389/fcvm.2021.668653

**Published:** 2021-05-20

**Authors:** Anna Chuda, Marcin Kaszkowiak, Maciej Banach, Marek Maciejewski, Agata Bielecka-Dabrowa

**Affiliations:** ^1^Heart Failure Unit, Department of Cardiology and Congenital Diseases of Adults, Polish Mother's Memorial Hospital Research Institute, Łódz, Poland; ^2^Department of Hypertension, Chair of Nephrology and Hypertension, Medical University of Lodz, Łódz, Poland; ^3^Department of Biostatistics and Translational Medicine, Medical University of Lodz, Łódz, Poland; ^4^Department of Cardiology and Congenital Diseases of Adults, Polish Mother's Memorial Hospital Research Institute, Łódz, Poland

**Keywords:** heart failure, atrial fibrillation, dehydration, body mass index, risk factors

## Abstract

**Purpose:** The aim of the study was to assess the relationship of dehydration, body mass index (BMI) and other indices with the occurrence of atrial fibrillation (AF) in heart failure (HF) patients.

**Methods:** The study included 113 patients [median age 64 years; 57.52% male] hospitalized due to HF. Baseline demographics, body mass analysis, echocardiographic results, key cardiopulmonary exercise test (CPET) parameters, 6 min walk distance (6MWD) and Kansas City Cardiomyopathy Questionnaire (KCCQ) score were assessed.

**Results:** Of all patients, 23 (20.35%) had AF, and 90 (79.65%) had sinus rhythm (SR). Patients with AF were older (med. 66 vs. 64 years; *p* = 0.039), with higher BMI (32.02 vs. 28.51 kg/m^2^; *p* = 0.017) and percentage of fat content (37.0 vs. 27.9%, *p* = 0.014). They were more dehydrated, with a lower percentage of total body water (TBW%) (45.7 vs. 50.0%; *p* = 0.022). Clinically, patients with AF had more often higher New York Heart Association (NYHA) class (III vs. II; *p* < 0.001), shorter 6MWD (median 292.35 vs. 378.4 m; *p* = 0.001) and a lower KCCQ overall summary score (52.60 vs. 73.96 points; *p* = 0.002). Patients with AF had significantly lower exercise capacity as measured by peak oxygen consumption (peak VO2) (0.92 vs. 1.26 mL/min, *p* = 0.016), peak VO2/kg (11 vs. 15 mL/kg/min; *p* < 0.001), and percentage of predicted VO2max (pp-peak VO2) (62.5 vs 70.0; p=0.010). We also found VE/VCO2 (med. 33.85 vs. 32.20; *p* = 0.049) to be higher and peak oxygen pulse (8.5 vs. 11 mL/beat; *p* = 0.038) to be lower in patients with AF than in patients without AF. In a multiple logistic regression model higher BMI (OR 1.23 per unit increase, *p* < 0.001) and higher left atrial volume index (LAVI) (OR 1.07 per unit increase, *p* = 0.03), lower tricuspid annular plane systolic excursion (TAPSE) (OR 0.74 per unit increase, *p* =0.03) and lower TBW% in body mass analysis (OR 0.90 per unit increase, *p* =0.03) were independently related to AF in patients with HF.

**Conclusion:** Increased volume of left atrium and right ventricular systolic dysfunction are well-known predictors of AF occurrence in patients with HF, but hydration status and increased body mass also seem to be important factors of AF in HF patients.

## Introduction

Atrial fibrillation (AF) is one of the most common arrhythmias worldwide. It currently affects over 6 million people in Europe and about 2.7–6.1 million people in the United States (1, 2). The incidence is projected to increase from around 7 million today to nearly 13 million individuals by 2030 and 15.9 million in 2050, with 120–215,000 new cases per year (3). AF is independently associated with increased morbidity and death from chronic diseases, including ischemic stroke (5-fold) (4), dementia and cognitive impairment (2-fold) (5, 6), heart failure (HF) (3-fold increase in the risk of HF and hospitalizations) (7, 8), and all-cause mortality (2-fold) (9–11). Data from the Framingham study (9, 11) demonstrated that HF and AF frequently coexist (about 20% with AF have HF, and about 5–50% with HF suffer with AF), and HF and AF frequently coexist, and their co-presence is associated with similar risk factors. (hypertension, smoking, obesity, diabetes mellitus, renal failure, sleep apnea, coronary artery disease) and similar pathophysiology [neurohormonal failure, activation of the renin–angiotensin–aldosterone system (RAAS), increased filling pressures and afterload, increased stretch, and fibrosis of the left atrium]. Pathophysiologic changes in the failing heart (the structural and neurohormonal changes) promote episodes of AF (about 1/3 of HF patients will develop AF) and promote AF from paroxysmal to permanent (12). The incidence of AF in the HF population rises with the New York Heart Association (NYHA) class (from 4% in NYHA class I to 50% in NYHA class IV) (12), while AF diagnosis in turn predisposes to the development of HF in the future (2- to 3-fold increased risk of incident HF) (8, 11, 12). Concomitant HF leads to higher mortality in AF subjects (8, 11). A potential explanation might be a particularly high risk of thromboembolism (through stasis, inflammation, and cellular dysfunction) in patients with both these diseases (11). However, AF may also have a negative impact on the daily life of HF patients due to reduced exercise tolerance (13, 14). Exercise dyspnea is a common complaint in patients with AF, and patients report a significant deterioration in the quality of life (15). Some studies have demonstrated improved quality of life and exercise capacity and/or symptom relief in AF patients when sinus rhythm (SR) has been restored (16, 17).

The aim of the study was to assess the relationship of dehydration, body mass index (BMI), and other indices with the occurrence of AF in HF patients and to assess the influence of this arrhythmia on exercise capacity assessed by the cardiopulmonary exercise testing (CPET) and 6-min walk test (6MWT) and to clarify the impact of AF on patients' quality of life assessed by the Kansas City Cardiomyopathy Questionnaire (KCCQ).

## Materials and Methods

This study was conducted in the Heart Failure Unit, Department of Cardiology and Congenital Heart Diseases of Adults in Polish Mother's Memorial Hospital Research Institute, in a tertiary hospital in the city of Lodz, Poland. The study followed the Declaration of Helsinki and was approved by Polish Mother's Memorial Hospital Research Institute (PMMHRI-BCO.16/2019). The ClinicalTrials.gov database is provided herein (No NCT04753814).

### Subject

The study included 113 HF patients {median age 64 years [interquartile range (IQR) 58–69]; 57.52% male}. Patients were divided into two groups:

1. Patients with long-standing AF (longer than 1 year) diagnosed according to the 2006 American College of Cardiology (ACC)/American Heart Association (AHA) and the European Society of Cardiology (ESC) Committee guidelines on AF (18); 23 patients [median age 66 years (IQR 62–73); 65.22% male].
2. Patients without a history of AF and with normal SR in electrocardiographic (ECG) analysis (non-AF); 90 patients [median age 64 years (IQR 54.8–68); 55.56% male].

In the next step, propensity score matching (PSM) was used to construct an artificial control group by matching AF participants one-to-one to participants without AF. Data were collected from 23 AF patients and 23 age-matched non-AF patients with HF.

### Eligibility Criteria

The study's inclusion and exclusion criteria are defined below.

#### Inclusion Criteria

- Age equal to or older than 18 years
- HF (ischemic and non-ischemic) diagnosed according to the 2016 ESC guidelines on HF (19), with HF class I, II, or III according to the NYHA classification (20)
- Current HF hospitalization
- Left ventricular ejection fraction (LVEF) documented in echocardiography during the current hospitalization

#### Exclusion Criteria

- Advanced liver failure [class B and C according to Child–Pugh score (21)]
- Advanced chronic kidney disease [stages G4 and G5 according to the 2012 Kidney Disease: Improving Global Outcomes (KDIGO) classification (22)]
- Cerebrovascular accident [transient ischemic attack (TIA)/stroke/intracerebral hemorrhage] within 3 months prior to hospitalization
- Current pregnancy or lactation
- Alcohol and drug abuse
- Active autoimmune disease
- Surgery or a serious injury within 1 month prior to hospitalization
- Other important medical conditions that could have shortened the survival time during the study
- Impaired cognitive status that compromises the understanding of the steps and completion of the study

### Protocol

Every patient underwent a clinical assessment. The following data were included in this analysis:

- baseline demographics (age and gender);
- medical history (comorbidities, HF etiology, and history of HF including HF hospitalization in the past);
- medication data [angiotensin-converting enzyme inhibitors (ACEIs); angiotensin receptor blockers (ARBs); beta blockers (BBs); mineralocorticoid receptor antagonists (MRAs); and diuretics];
- assessment of the severity of HF symptoms according to the NYHA classification (20);
- physical examination [peripheral hypoperfusion, hepatocervical symptom, edema, lung congestion, third heart sound (S3), and liver enlargement].

In all patients, the following diagnostic procedures were also performed:

- selected laboratory tests on admission [N-terminal prohormone brain natriuretic peptide (NT-proBNP); high-sensitivity cardiac troponin T (hs-cTnT); C-reactive protein (CRP); hemoglobin; sodium; potassium; creatinine; and uric acid];
- KCCQ score;
- bioelectrical impedance body mass analysis;
- Doppler echocardiography;
- ECG;
- CPET;
- 6MWT;
- concentration of selected biomarkers [neprilysin, galectin-3, high-sensitivity CRP (hs-CRP), and suppression of tumorigenicity 2 (ST-2)].

### Measurements

#### Doppler Echocardiography

For the clinical and diagnostic stratification, all of the 113 HF patients were submitted to a two-dimensional (2D) echocardiogram using a Vivid E95 system (GE Healthcare, Chicago, USA) with a 1.4–5.2 MHz matrix transducer and tissue Doppler imaging software. Body surface area (BSA) was obtained using DuBois and Dubois' formula (23). The echocardiographic measurements were performed according to the guidelines of the American Society of Echocardiography (ASE) and European Association of Echocardiography (EAE) (24). Echocardiographic assessment was performed on all patients including M-mode and 2D images. The following echocardiographic parameters were included in this analysis: LVEF, left ventricular end-diastolic diameter (LVDD), left ventricular end-systolic diameter (LVESD), left ventricular end-diastolic volume (LVEDV), left ventricular end-systolic volume (LVESV), left atrial volume index (LAVI), tricuspid annular plane systolic excursion (TAPSE), right ventricular systolic pressure (RVSP), and inferior vena cava (IVC) size. LVEF was calculated using Simpson's method (24). HF with preserved ejection fraction (HFpEF) was defined by an LVEF ≥ 50%. HF with reduced ejection fraction (HFrEF) was diagnosed if LVEF was <40%, and HF with mid-range ejection fraction (HFmrEF) was diagnosed if LVEF was 40–49% (19). Manual tracings of left ventricular endocardial borders were obtained at end-diastole and end-systole in the apical views, and respective volumes were derived using the modified biplane Simpson rule or Teichholz method, depending on image quality. Left atrial volume was obtained using the biplane area-length method from apical views and indexed to BSA. TAPSE was averaged over five cardiac cycles. RVSP was acquired as a measure of pulmonary artery (PA) pressure. RVSP was estimated based on the average peak gradient of tricuspid regurgitant jets from 5 cycles and adding the estimated RA pressure based on the IVC diameter and collapse. Routine measurements in size of the IVC and collapsibility with respiration were measured using 2D or M-mode assessment of IVC (24, 25).

#### Electrocardiographic Data

Standard 12-lead ECGs were recorded using MAC 5500 machines (GE Medical Systems, Milwaukee, USA). Every ECG was recorded on admission. With the initial visual inspection, recordings with inadequate quality were excluded. The MAC 5500 machines measured the QRS duration from the beginning of the Q wave to the end of the S wave in all 12 leads. The MAC 5500 machines measured the QT interval from the onset of the QRS complex to the end of the T wave in all 12 leads. The averaged QT interval was then corrected for heart rate (HR) using the Bazett formula (QTc = QT/√RR).

#### Body Mass Analysis

Bioelectrical impedance analysis (BIA) was performed with the body composition analyzer MC-780MA S (Tanita, Amsterdam, Netherlands), which performs 15 impedance measurements per patient, by measuring conductance of electrical current across five body segments (legs, arms, and whole body) at three frequencies each (5, 50, and 250 kHz). The following parameters were included in this analysis: weight, BMI, fat %, fat mass (FM), fat-free mass (FFM), total body water (H_2_O-TBW), extracellular water (ECW), intracellular water (ICW), ECW ratio normalized for H_2_O-TBW (ECW/TBW), and metabolic age (MA).

#### Cardiopulmonary Exercise Testing

Symptom-limited CPET was performed on an electromagnetically braked upright cycle ergometer Bike M (CORTEX Biophysik GmbH, Leipzig, Germany) with a metabolic gas analyzer METALYZER 3B (CORTEX Biophysik GmbH, Leipzig, Germany) using The MetaSoft Studio application software of CORTEX systems. The system was calibrated with a standard gas mixture of known concentrations before each test. The breath gas analyzer was internally calibrated directly prior to each measurement. The volume of gases and the flow sensor were also calibrated directly prior to the test, validating the calibration twice a year.

After 5 min of rest on the cycle ergometer, exercise commenced at 50 W, then, the work rate was increased by 25 W every 3 min. During CPET, blood pressure was measured by a conventional sphygmomanometer Exacta (Rudolf Riester GmbH, Jungingen, Germany) every 3 min. HR and standard 12-lead ECG were monitored using an exercise ECG Meta control 3000 (CORTEX Biophysik GmbH, Leipzig, Germany), which connects the CORTEX METALYZER 3B. The criteria for discontinuation of CPET were as follows:

- maximum HR of 220 bpm minus years of age;
- drop in HR during exercise;
- drop in systolic blood pressure (SBP) of >10 mmHg from baseline blood pressure despite an increase in workload;
- signs of poor perfusion (cyanosis or pallor);
- ST segment depression (>1.0 mm) or elevation (>1.0 mm);
- lack of ventilatory reserve;
- respiratory exchange rate (RER) higher than 1.15;
- plateau in consumption of oxygen, with an increase of <150 mL/min in about 30 s;
- personal discomfort (maximal fatigue, thoracic pain, severe dizziness, near syncope, excessive dyspnea, and subject signals to examiner to end the examination);
- severe cardiac arrhythmia (progressive atrial or ventricular arrhythmia or block images, newly occurring AF).

Expired gases were continuously measured in all subjects on a breath-by-breath basis. Uptake of oxygen was measured in breath-by-breath fashion, recording mean values over 30 s.

Before, during, and after the test, we recorded continuously HR, breathing frequency, minute volume, uptake of oxygen, and elimination of carbon dioxide. Blood gas analyses were recorded at rest (rest; average of 5 min of rest on the cycle ergometer), at anaerobic threshold (AT), at the exercise peak (Peak), and 90 s after the end of the effort.

The RER was defined as the ratio between carbon dioxide production (VCO_2_) and oxygen production (VO_2_). A peak RER of >1.10 was considered an indication of excellent subject effort during CPET.

Peak VO_2_ (VO_2_max) was defined as the mean of values measured within the last 20 s of exercise and expressed as both mL/min and mL/kg/min (VO_2_max/kg) and as the percentage of predicted peak oxygen consumption (VO_2_%Norm).

AT was determined by gas-exchange criteria as the point of non-linear increase in ventilation equivalents for oxygen (ventilation starts to increase at a faster rate than VO_2_).

The minute ventilation/carbon dioxide production relation slope (VE/VCO_2_ slope), the slope of the increase in ventilation to the increase in CO_2_ output, was calculated during incremental exercise using least squares linear regression by the computer system METALYZER 3B.

O_2_ pulse (VO_2_/HR) was defined by dividing VO_2_ by HR.

VO_2_%Norm at AT and Peak were calculated according to published age and sex-normalized values (26).

#### 6-Min Walk Test

The walking tests were conducted according to the standardized protocol (27). Patients were asked to walk as far as possible in 6 min along a 30-m long corridor. Standardized instructions and encouragement were commonly given during the test. The test was finished when patients were unable to continue the test or when they were desaturated. The distance walked at the end of 6 min (6MWTD) was recorded in meters.

#### Quality of Life

The KCCQ was used to assess the quality of life of the patients. It is a validated instrument to assess health status and quality of life among persons with HF. The self-administered questionnaire includes 23 items, which quantify the importance of dyspnea, fatigue, and edema for physical, social, and emotional functions. Patients answer the questions as they related to the previous 2 weeks. An overall summary score can be derived from the physical function, symptom (frequency and severity), social function, and quality of life domains. For each domain, the validity, reproducibility, responsiveness, and interpretability have been independently established. Scores are transformed to a range of 0–100, in which higher scores reflect better health status (28).

#### Laboratory Analysis

Approximately 10 ml of blood from a peripheral vein was collected into a tube containing potassium ethylenediamine tetra-acetic acid (1 mg/mL) for the determination of selected biomarkers. The samples were centrifuged at 4°C for 20 min. The plasma was separated and subsequently frozen at −70°C until further analysis without undergoing any additional freeze–thaw cycles. Concentrations of biomarkers were measured by dedicated ELISA immunoassays.

### Statistical Analysis

Statistical analysis was conducted using the R language (4.02). All continuous variables were expressed as median and IQR (Q25–Q75). Categorical variables were expressed as the number of observations (N) and the corresponding percentage (%). The Mann–Whitney U test was used to compare continuous variables. The χ^2^ test was used to compare the qualitative data between the groups. Fisher's exact test for independence (in lower numbers *N* < 5) was used. All statistical tests were two-sided. Results were considered statistically significant when *p* < 0.05. The tables present only variables that differ or are selected in the context of the article.

Logistic regression was used to evaluate the impact of the factors on AF occurrence, in both univariate and multivariate designs. The odds ratio (OR) and 95% confidence interval (95% CI) were reported for each factor in univariate and multivariate analyses. All variables significantly associated (*p* < 0.05) with AF occurrence in the univariate model were included in the multivariate regression analysis to determine the independent predictors of the AF occurrence. Tables with the results of univariate and multivariate analyses show only statistically significant data. PSM was used to construct an artificial control group by matching each AF unit with a non-AF unit of similar characteristics. Due to a high imbalance between genders in group with AF on admission, correctly performing PSM on both sex and age was not possible. Therefore, we have decided to use only age in PSM. Data were collected from 23 AF patients and 23 age-matched non-AF patients. Using these matches, the researchers could estimate the impact of the factors on AF occurrence.

## Results

### Evaluation of Basic Characteristics of the Studied Groups

Based on the available echocardiography results (*N* = 113), the mean LVEF was 44.0% (IQR 39.0–50.0). Of those patients, 26.55% (*N* = 30) had HFpEF, 46.90% (*N* = 53) had HFmrEF, and 26.55% (*N* = 30) had HFrEF. Ischemic etiology of HF was present in 43.36% of all patients (*N* = 49). Of all patients with baseline rhythm data, 23 (20.35%) had AF, and 90 (79.65%) had SR in ECG. The studied population in both groups was burdened with the most common comorbidities including arterial hypertension, coronary artery disease, diabetes mellitus, valve diseases, and hyperlipidemia. Detailed characteristics of the study population are presented in Table 1. Prior to PS matching, there were no statistically significant differences in sex between AF and SR groups [15 men (65.22%) vs. 50 men (55.56%), *p* = 0.403]. No significant differences were also observed with regard to systolic and diastolic blood pressure between the SR and AF groups (Table 1). Patients with AF were older (med. 66 vs. 64 years; *p* = 0.039), with higher NYHA class (III vs. II; *p* < 0.001) and lower KCCQ overall summary score (52.60 vs. 73.96 points; *p* = 0.002) (Table 1). In ECG, the AF group had a higher HR (med. 80 vs. 70 bpm, *p* = 0.004), wider QRS (med. 112 vs. 98 ms; *p* = 0.004), and longer QTc (med. 467.5 vs. 437 ms; *p* = 0.005) duration (Table 1).

**Table 1:** General characteristics of study groups divided according to cardiac rhythm.

**Total**		**Subgroup**		
		**Sinus rhythm**	**Atrial fibrillation**	***p***
All patients, *N* (%)	113 (100)	90 (79.65)	23 (20.35)	
Males, *N* (%)	65 (57.52%)	50 (55.56)	15 (65.22)	0.403
Age (years)	64.000 (58.0–69.0)	64.0 (55.00–68.00)	66.0 (62.00–73.00)	**0.039**
Body surface area (BSA) (m^2^)	1.949 (1.782–2.132)	1.935 (1.71–2.11)	2.049 (1.88–2.33)	**0.034**
SBP (mmHg)	134.0 (120.0–146.0)	135 (120–147)	130 (124–135)	0.138
DBP (mmHg)	80.0 (70.0–86.0)	80 (70–86)	83 (70–88)	0.276
Heart rate on admission (bpm)	70.0 (65.0–80.0)	70 (64–77)	80 (67–90)	**0.004**
Width of the QRS complex (ms)	99 (84–121.5)	98 (84–116)	112 (96–136)	**0.004**
QTcBAz (ms)	442 (425–475)	437 (424–462)	467.5 (437–494.5)	**0.005**
Ischemic etiology, *N* (%)	48 (42.48%)	43 (47.78)	5 (21.74)	**0.024**
Non-ischemic etiology, *N* (%)	65 (57.52%)	47 (52.22)	18 (78.26)	**0.024**
NYHA class on admission	2 (2.0–2.5)	2 (2.0–2.0)	3 (2.5–3.0)	**<0.001**
6MWT distance (m)	370.5 (290.7–449.2)	378.4 (306.1–457.0)	292.35 (201.3–349.3)	**0.001**
KCCQ score (point)	70.05 (50.0–83.33)	73.96 (57.29–83.98)	52.60 (38.28–71.15)	**0.002**
Coronary artery disease, *N* (%)	53 (46.90)	47 (52.2)	6 (26.09)	**0.025**
Mitral regurgitation, *N* (%)	55 (48.67%)	39 (43.33)	16 (69.57)	**0.025**
Aortic regurgitation, *N* (%)	16 (14.16%)	11 (12.22)	5 (21.74)	0.243
Tricuspid regurgitation, *N* (%)	40 (35.40%)	25 (27.78)	15 (65.22)	**0.001**
Aortic stenosis, *N* (%)	5 (4.42%)	3 (3.33)	2 (8.69)	0.268
Diabetes mellitus, *N* (%)	31 (27.43)	22 (24.44)	9 (39.13)	0.159
Arterial hypertension, *N* (%)	83 (73.45)	61 (67.78)	22 (95.65)	**0.007**
Hyperlipidemia, *N* (%)	86 (76.11%)	69 (76.67)	17 (73.91)	0.782
ACEI, *N* (%)	75 (66.37)	60 (66.67)	15 (65.22)	0.896
ARB, *N* (%)	18 (15.93)	13 (14.44)	5 (21.74)	0.522
BB, *N* (%)	98 (86.73)	78 (86.67)	20 (86.96)	1.00
MRA, *N* (%)	52 (46.02)	39 (43.33)	13 (56.52)	0.257
Diuretics, *N* (%)	65 (57.52)	47 (52.22)	18 (78.26)	**0.033**

*Data are given as median (interquartile range) unless otherwise indicated. A p-value of <0.05 is considered statistically significant*.

*DBP, diastolic blood pressure; SBP, systolic blood pressure; QTcBAz, QT interval corrected for heart rate; NYHA, New York Heart Association; 6MWT, 6-min walk test; KCCQ, Kansas City Cardiomyopathy Questionnaire; ACEI, angiotensin-converting enzyme inhibitors; ARB, angiotensin receptor blockers; BB, beta blockers; MRA, mineralocorticoid receptor antagonists. Bold values are statistically significant data*.

After PS matching, the 23 matched pairs were analyzed for differences in the baseline characteristics (Table 2). There were no significant discrepancies in the overall characteristics of the groups after PS matching compared with the previous analysis (Table 2).

**Table 2:** Clinical characteristics of patients in the AF and non-AF groups after PS matching.

	**Sinus rhythm**	**Atrial fibrillation**	***p***
All patients (*N*)	23	23	
Age (years)	66.0 (62.00–73.00)	66.0 (62.00–73.00)	0.965
SBP (mmHg)	137 (127.3–149.0)	130 (124–135)	0.102
DBP (mmHg)	75 (70–82.75)	83 (70–88)	0.199
Heart rate on admission (bpm)	69 (60–70.75)	80 (67–90)	**0.002**
Width of the QRS complex (ms)	92 (84–134)	112 (96–136)	**0.047**
QTcBAz (ms)	431.5 (415.75–451)	467.5 (436.5–494.8)	**0.006**
NYHA class on admission	2 (2.0–3.0)	3 (2.5–3.0)	**0.013**
6MWT distance (m)	378.4 (318–459.7)	292.35 (201.25–357.68)	**0.005**
KCCQ score (point)	69.80 (55.47–84.05)	52.60 (38.28–71.15)	**0.015**

*Data are given as median (interquartile range) unless otherwise indicated. A p-value of <0.05 is considered statistically significant*.

*DBP, diastolic blood pressure; SBP, systolic blood pressure; QTcBAz, QT interval corrected for heart rate; NYHA, New York Heart Association; 6MWT, 6-min walk test; KCCQ, Kansas City Cardiomyopathy Questionnaire. Bold values are statistically significant data*.

Regarding the cardiovascular background, the proportion of patients with coronary artery disease (52.2 vs. 26.09%; *p* = 0.025) and ischemic etiology of HF (47.78 vs. 21.74%; *p* = 0.024) was higher in the SR group, whereas the proportion of patients with hypertension (95.65 vs. 67.78%; *p* = 0.007) and with valvular disease, i.e., mitral regurgitation (69.57 vs. 43.33%; *p* = 0.025) and tricuspid regurgitation (65.22 vs. 27.78%; *p* = 0.001), was higher in the AF group (Table 1). There were no significant differences in the use of renin–angiotensin system blockers (ACEI and ARB), BB, or MRA between AF and SR groups (Table 1). However, diuretics were used more frequently in the AF group (78.26 vs. 52.22%; *p* = 0.033) (Table 1).

### Evaluation of Echocardiographic Parameters

AF group had greater LAVI (med. 65 vs. 40 mL/m^2^; *p* < 0.001) and LVDD (med. 61 vs. 54 mm; *p* < 0.001) than had the SR group. TAPSE was lower (med. 18 vs. 22 mm; *p* < 0.001) and RVPs were higher in the AF group than in the SR group (med. 38 vs. 28 mmHg; *p* < 0.001) (Table 3). There were no statistically significant differences in LVEF between the AF and SR groups (Table 3).

**Table 3:** Structural echocardiographic variables in the AF and non-AF groups.

	**Sinus rhythm**	**Atrial fibrillation**	***p***
LVEF (%)	44.50 (39.0–55.0)	41.00 (36.0–45.0)	0.133
TAPSE (mm)	22.0 (19.0–24.0)	18.0 (16.0–20.0)	**<0.001**
LVDD (mm)	54 (49–62)	61 (55–65)	**0.047**
LVSD (mm)	38 (32–48)	44 (34–51)	0.129
LVEDV (cm^3^)	124 (91–159)	116 (93–221)	0.679
LVESV (cm^3^)	65.5 (40–95)	65 (57–129)	0.237
VCI (mm)	16.5 (13.0–19.0)	19.0 (14.0–21.0)	0.114
RVPs (mmHg)	28 (25–33)	38 (31–51.5)	**<0.001**
LAVI (mL/m^2^)	40 (30.4–47.60)	65 (44.49–74)	**<0.001**

*Data are given as median (interquartile range) unless otherwise indicated. A p-value of <0.05 is considered statistically significant*.

*LVEF, left ventricle ejection fraction; TAPSE, tricuspid annular plane systolic excursion; LVDD, left ventricular diastolic diameter; LVSD, left ventricular systolic diameter; LVEDV, left ventricular end-diastolic volume; LVESV, left ventricular end-systolic volume; VCI, inferior vena cava; RVPs, right ventricular systolic pressure; LAVI, left atrial volume index. Bold values are statistically significant data*.

### Evaluation of Exercise Capacity

Median VO_2_max (0.92 vs. 1.26 L/min, *p* = 0.016), VO_2_max/kg (11 vs. 15 mL/min/kg; *p* < 0.001), and VO_2_%Norm (62.5 vs. 70.0%; *p* = 0.010) were significantly lower in the AF group vs. the non-AF group (Table 4). RER in the study population was 1.080 (IQR 0.98–1.14) and was lower in the AF than in the non-AF group (*p* = 0.001) (Table 4). VO_2_ at AT was also lower in the AF group (0.735 vs. 1.00 L/min; *p* = 0.033) (Table 4). We also found VE/VCO_2_ to be higher in patients with AF vs. patients without AF (med. 33.85 vs. 32.20; *p* = 0.049) (Table 4). Moreover, VO_2_/HR at peak (8.5 vs. 11 mL/beat; *p* = 0.038) was significantly impaired in the AF group (Table 4). The other differences in characteristics were not statistically significant and are presented in Table 4.

**Table 4:** Analysis of CPET variables in AF and non-AF groups.

	**Sinus rhythm**	**Atrial fibrillation**	***p***
RER	1.090 (1.00–1.14)	0.965 (0.94–1.05)	**0.001**
Predicted VO_2_max (%)	70.0 (58.0–81.0)	62.5 (45.0–68.0)	**0.010**
VO_2_max (L/min)	1.26 (0.98–1.68)	0.92 (0.7–1.2)	**0.016**
VO_2_max/kg (mL/min/kg)	15 (12–19)	11 (9–14)	**<0.001**
VO_2_ AT (L/min)	1.00 (0.76–1.19)	0.735 (0.67–0.96)	**0.033**
VE/VO_2_	32.70 (29.6–39.6)	34.55 (30.9–39.8)	0.655
VE/VCO_2_	32.20 (27.3–35.3)	33.85 (31.2–41.1)	**0.049**
VCO_2_ slope	31.2 (26.0–37.2)	34.6 (30.9–39.8)	0.052
VO_2_/HR peak (mL/bpm)	11 (9–13)	8.5 (7–12)	**0.038**

*Data are given as median (interquartile range) unless otherwise indicated. A p-value of <0.05 is considered statistically significant*.

*RER, respiratory exchange ratio; VO_2_max, maximal oxygen consumption; VO_2_, oxygen uptake; AT, anaerobic threshold; VE, minute ventilation; VCO_2_, CO_2_ output; VE/VO_2_, ventilatory equivalent for O_2_; VE/VCO_2_, ventilatory equivalent for CO_2_; VO_2_/HR, peak VO_2_ divided by the heart rate. Bold values are statistically significant data*.

Furthermore, compared with patients in SR, patients with AF had significantly lower exercise capacity measured as 6MWTD (med. 292.35 vs. 378.4 m; *p* = 0.001) (Table 1).

### Evaluation of Body Composition

Patients with AF had a significantly higher BMI (med. 32.02 vs. 28.51 kg/m^2^, *p* = 0.017) than their counterparts (Table 5). Moreover, compared with the SR group, the patients with AF had significantly higher BIA parameters, including FM (med. 30.07 vs. 22.50 kg; *p* = 0.007), FM% (med. 37.0 vs. 27.9%; *p* = 0.014), ECW (med. 18.5 vs. 18.2 kg; *p* = 0.031), and MA (med. 66 vs. 59 years; *p* = 0.001), and a lower H_2_O-TBW% (45.7 vs. 50.0%; *p* = 0.022) (Table 5). The other differences in characteristics were not statistically significant and are presented in Table 5.

**Table 5:** Comparison between body mass analysis variables in AF and non-AF groups.

	**Sinus rhythm**	**Atrial fibrillation**	***p***
Body mass index (kg/m^2^)	28.51 (25.29–32.45)	32.02 (27.68–38.3)	**0.017**
FM% (%)	27.90 (23.8–36.5)	37.00 (24.5–41.7)	**0.014**
FM (kg)	22.5 (16.2–32.6)	30.07 (19.3–55.3)	**0.007**
FFM (kg)	57.50 (47.2–68.6)	60.60 (52.3–77.3)	0.137
H_2_O-TBW (%)	50.00 (45.1–53.5)	45.70 (44.2–50.4)	**0.022**
H_2_O-TBW (kg)	40.10 (33.4–47.4)	42.30 (36.7–58.7)	0.090
ECW (kg)	18.20 (15.3–20.2)	18.50 (17.2–26.0)	**0.031**
ICW (kg)	21.30 (18.3–27.3)	23.05 (19.3–32.7)	0.233
ECW/TBW (%)	44.20 (42.5–46.3)	44.40 (44.3–47.4)	0.125
MA (years)	59 (47–68)	66 (58–79)	**0.001**

*Data are given as median (interquartile range) unless otherwise indicated. A p-value of <0.05 is considered statistically significant*.

*FM, body fat mass; FM%, percentage of fat mass; FM, fat mass; FFM, fat-free mass; H_2_O-TBW, total body water; ECW, extracellular water; ICW, intracellular water; ECW/TBW extracellular water ratio normalized for total body water; MA, metabolic age. Bold values are statistically significant data*.

### Evaluation of Selected Biochemical Biomarkers

Patients with AF showed a higher concentration of sodium (med. 142 vs. 141 mmol/L, *p* = 0.020) (Table 6) and uric acid (med. 7.10 vs. 6.05 mg/dl, *p* = 0.001) and a significantly higher level of biomarkers associated with inflammation (CRP and hs-CRP) and myocyte stress and injury (NT-proBNP and hs-cTnT) than non-AF individuals (Table 6). The other characteristics were similar between the groups (Table 6).

**Table 6:** Analysis of laboratory tests and biomarkers results in AF and non-AF groups.

	**Sinus rhythm**	**Atrial fibrillation**	***p***
NT-proBNP (pg/mL)	317.0 (190.5–649.5)	1,363.0 (483.0–2,360.0)	**<0.001**
hs-cTnT (pg/mL)	9.6 (7.3–15.0)	22.3 (11.5–26.9)	**0.015**
Hemoglobin (g/dl)	13.40 (12.3–14.5)	13.80 (12.7–14.9)	0.608
Sodium (mmol/L)	141 (140.0–142.0)	142 (140.0–143.0)	**0.020**
Potassium (mmol/L)	4.4 (4.2–4.6)	4.5 (4.3–4.7)	0.506
Creatinine (μmol/L)	0.87 (0.71–1.02)	0.97 (0.78–1.21)	0.130
CRP (mg/L)	0.50 (0.5–0.77)	0.83 (0.5–1.62)	**0.015**
Uric acid (mg/dl)	6.05 (5.15–7.20)	7.10 (6.10–8.30)	**0.001**
Neprilysin (pg/mL)	458.628 (301.45–840.22)	437.927 (296.26–797.38)	0.88
Galectin-3 (ng/mL)	9.55 (7.54–13.02)	10.404 (7.58–13.72)	0.522
hs-CRP (μg/mL)	1.91 (0.90–3.82)	5.24 (2.71–10.86)	**0.002**
ST-2 (pg/mL)	32.40 (27.65–44.52)	66.37 (37.71–118.38)	0.207

*Data are given as median (interquartile range) unless otherwise indicated. A p-value of <0.05 is considered statistically significant*.

*NA, perfect separation detected results not available*.

*NT-proBNP, N-terminal prohormone brain natriuretic peptide; hs-cTnT, high-sensitivity cardiac troponin T; CRP, C-reactive protein; hs-CRP, high-sensitivity C-reactive protein; ST-2, suppression of tumorigenicity 2. Bold values are statistically significant data*.

### Univariate and Multivariate Analyses

Prior to matching, the univariate regression analysis revealed the significant variables affecting AF occurrence in HF patients (Table 7). A few significantly associated parameters were then used in the multivariate regression analysis. A multiple logistic regression model showed that increased body mass, with higher BMI (OR 1.23 per unit increase; 95% CI: 1.09–1.46; *p* < 0.001), enlargement of the left atrium, greater LAVI (OR 1.07 per unit increase; 95% CI: 1.04–1.14; *p* = 0.03), lower TAPSE (OR 0.74 per unit increase; 95% CI: 0.46–0.81; *p* = 0.03), and lower H_2_O-TBW% (OR 0.90 per unit increase; 95% CI: 0.81–0.98; *p* = 0.03) were independently related to AF in patients with HF (Table 7).

**Table 7:** Univariate and multivariate analyses of association of patient characteristics with the occurrence of AF before PS matching.

**Variables**	**Exp(B)–OR**	**95% CI for OR**		***p***
**Univariate analysis**				
Echocardiography parameters				
TAPSE (mm)	0.77	0.65	0.87	<0.001
RVPs (mmHg)	1.15	1.07	1.26	<0.001
LAVI (ml/m^2^)	1.06	1.03	1.10	<0.001
Body mass analysis results				
Body mass index (kg/m^2^)	1.12	1.04	1.22	0.01
FM% (%)	1.08	1.02	1.16	0.01
FM (kg)	1.06	1.02	1.10	<0.001
H_2_O-TBW (%)	0.90	0.81	0.99	0.03
ECW (kg)	1.17	1.03	1.34	0.02
MA (years)	1.05	1.02	1.10	0.01
Laboratory tests results				
NT-proBNP (pg/mL)	1.00	1.00	1.00	0.001
hs-cTnT (pg/mL)	1.04	1.00	1.08	0.054
CRP (mg/L)	1.74	1.06	3.09	0.036
Uric acid (mg/dl)	1.61	1.20	2.21	0.002
hs-CRP (μg/mL)	1.23	1.07	1.43	0.004
**Multivariate analysis**				
Body mass index (kg/m^2^)	1.23	1.09	1.46	<0.001
TAPSE (mm)	0.64	0.46	0.81	<0.001
LAVI (mL/m^2^)	1.08	1.04	1.14	<0.001
H_2_O-TBW (%)	0.90	0.81	0.98	0.03

*The table shows only statistically significant data, with a p-value of <0.05*.

*TAPSE, tricuspid annular plane systolic excursion; RVPs, right ventricular systolic pressure; LAVI, left atrial volume index; FM%, percentage of fat mass, FM, fat mass FFM, fat-free mass; H_2_O-TBW, total body water; ECW, extracellular water; MA, metabolic age; NT-proBNP, N-terminal prohormone brain natriuretic peptide; hs-cTnT, high-sensitivity cardiac troponin T; CRP, C-reactive protein; hs-CRP, high-sensitivity C-reactive protein*.

We estimated the impact of these factors on AF occurrence in univariate and multivariate regression models also after PS matching (Table 8). The univariate regression analysis revealed similar variables affecting AF occurrence in HF patients compared with results before PS matching (Table 8). Only the laboratory results showed differences, and the uric acid and troponin T were the only parameters significantly associated with AF after PS matching. In a multiple logistic regression model after PS matching, only an increase of AF occurrence was significantly associated with greater LAVI (OR 1.091; 95% CI: 1.036–1.172; *p* = 0.004). The other variables were similar compared with results before PS-matching analysis, but not statistically significant (Table 8).

**Table 8:** Univariate and multivariate analyses of association of patient characteristics with the occurrence of AF after PS matching.

**Variables**	**Exp(B)–OR**	**95% CI for OR**		***p***
**Univariate analysis**				
Echocardiography parameters				
TAPSE (mm)	0.71	0.56	0.86	<0.001
RVPs (mmHg)	1.21	1.07	1.50	0.02
LAVI (mL/m^2^)	1.06	1.02	1.11	<0.001
Laboratory tests results				
hs-cTnT (pg/mL)	1.10	1.02	1.19	0.02
Uric acid (mg/dl)	1.60	1.09	2.59	0.03
**Multivariate analysis**				
LAVI (mL/m^2^)	1.091	1.036	1.172	0.004

*The table shows only statistically significant data, with a p-value of <0.05*.

*TAPSE, tricuspid annular plane systolic excursion; RVPs, right ventricular systolic pressure; LAVI, left atrial volume index; hs-cTnT, high-sensitivity cardiac troponin T*.

## Discussion

In this study, we compared patients with HF with accompanying AF with a group of HF patients with SR. We found that clinically patients with AF had a higher NYHA class, a shorter 6MWTD, and a lower KCCQ overall summary score (Table 1). Moreover, among the patients with HF, those with AF had significantly lower exercise tolerance, reflected in lower VO_2_max, VO_2_max/kg, VO_2_%Norm, and VO_2_/HR. They also had impaired ventilatory efficiency (higher VE/VCO_2_ slope) and lower submaximal exercise capacity (VO_2_ AT) (Table 4). Exercise intolerance, lower functional capacity, and lower quality of life in these patients result in a gradual increase in body weight. Consequently, this group had higher BMI, higher fat content (FM and FM%), and higher ECW in the body fluid composition (Table 5), so they were treated with diuretics more often (Table 1). As a result, they were paradoxically often more dehydrated, with lower TBW%, higher creatinine concentration, and higher sodium level (Tables 5, 6). They also had a significantly higher level of biomarkers associated with inflammation (CRP and hs-CRP) and myocyte stress and injury (NT-proBNP and hs-cTnT) than non-AF individuals (Table 6). We performed multifactorial analysis, which showed that higher BMI (OR 1.23 per unit increase), greater LAVI (OR 1.07 per unit increase), lower TAPSE (OR 0.74 per unit increase), and lower TBW% in body mass analysis (OR 0.90 per unit increase) were independently related to AF in patients with HF (Table 7). In a multiple logistic regression model after PS matching in relation to age and sex, only an increase of AF occurrence was significantly associated with greater LAVI (OR 1.091 per unit increase). The other variables were similar compared with results before PS-matching analysis, but not statistically significant (Table 8).

### Influence of Atrial Fibrillation on Parameters of Exercise Capacity in Heart Failure Patients

The 20% incidence of AF in our population is similar to that previously reported in HF population (29, 30). Numerous previous studies have demonstrated that AF is associated with exercise intolerance, mainly in patients with associated heart disease (31–33). In our study, compared with patients in SR, patients with AF had significantly lower exercise capacity measured by 6MWTD (med. 292.35 vs. 378.4 m; *p* = 0.001). Similarly, results were obtained by Fung et al. (33) (mean 6MWTD in AF 279 ± 66 vs. 338 ± 86 m in SR) and in the RELAX study (32) (mean 6MWTD in AF 283 ± 107 vs. 313 ± 109 in SR).

Other studies reported that peak VO_2_ is 10–20% lower in patients with HFrEF (14, 31) and significantly reduced in HFpEF (16, 32) patients with AF. Our research work confirmed these results. In our analysis, HF patients with AF had impaired peak exercise tolerance, reflected in lower VO_2_, lower VO_2_/kg, and lower VO_2_/HR at peak exercise as compared with those in the SR group. The lower cardiac output during AF due to loss of atrial contraction, irregular ventricular rhythm, and impaired ventricular filling is the most important during the peak of exercise when maximum systolic reserve is used (34). Linde-Edelstam et al. (35) reported that the significance of atrial contraction in LV filling decreases with increasing blood flow velocity and with increasing workload. Moreover, the pressure in the left atrium determines the share of atrial contraction in the LV filling, and an increase in the wedge pressure in pulmonary capillaries reduces the power of atrial contraction on the cardiac output (35).

Moreover, AF was associated in our study with impaired ventilatory efficiency (higher VE/VCO_2_). The mean VE/VCO_2_ slope was significantly higher in HF patients with AF compared with patients in SR efficiency also in previous studies (31, 32, 36).

Unexpectedly, we observed in our HF patients with AF compared with SR patients lower peak VO_2_ at AT, which is in contrast with a few previous reports (31, 36). The paradox of a lower VO_2_ at AT in our study could be related to the lower chronotropic response to exercise in our patients resulting from chronic use of BBs, also during hospitalization. This observation raises uncertainties about the use of AT data to define performance and prognosis of HF patients with AF supporting the need of multiparametric evaluation of CPET in this disease. In a study of Agostoni et al. (31), among all variables examined at AT, only HR was independently associated with AF. In AF, the increased HR response during exercises is necessary to maintain cardiac output; and the correlation between VO_2_ and cardiac output in HF is particularly strong at AT (31). Lewis et al. (37) demonstrated that a reduction in the ventricular rate in AF was associated with a small increase in stroke volume but was offset by rate-related reductions in cardiac output during exercise.

### Body Mass Index and Total Body Water % as Parameters Independently Associated With Atrial Fibrillation Occurrence in Heart Failure Patients

Compared with the SR group, the patients with AF in our study had a significantly greater BMI and higher body FM in BIA. The links between higher BMI and the risk of the new onset AF have been evidenced previously (overweight increased risk by 18–54% and obesity increased risk by 1.6- to 2.4-fold) (38, 39). There was a 28% increase in the relative risk per 5-unit increase in BMI, a 9% increase per 5-kg increase in body FM, and a 10% increase per 5-kg increase in body weight (39). In addition, REVERSE-AF (39) study has found that weight loss in overweight and obese individuals is associated with a reduced burden and reversal of AF. The mechanisms by which obesity increases the risk of cardiovascular diseases (CVDs) include changes in body composition [increases the percentage of epicardial adipose tissue (EAT)], which can affect hemodynamics and changes in the structure of the heart (left size enlargement and fibrosis, atrial inflammation and lipid infiltration, and changes in atrial electrophysiological properties) (40). Pro-inflammatory cytokines produced by adipose tissue could induce cardiac dysfunction and promote the formation of atherosclerotic plaques. When obesity and coronary heart disease (CHD) coexist, people with class I obesity have a better prognosis than those with normal weight or underweight. This phenomenon has been dubbed the “obesity paradox” (41). But this effect on prognosis in AF patients is controversial. In a study of elderly outpatients with AF, being underweight has been related to worse outcomes (42). On the other hand, in FANTASIIA Registry (43), obesity was associated with more comorbidities, but not with poor prognosis. Further studies are needed to determine the importance of obesity for the AF prognosis in patients with HF.

In our study, patients with AF were more likely to be older and more often were treated with diuretics, and in consequence, they were more dehydrated, with lower H_2_O-TBW% (*p* = 0.022), higher sodium level (*p* = 0.02), and higher creatinine concentration, but not statistically significant (*p* = 0.130). The lower H_2_O-TBW% was also independently related to AF presentation in our HF patients. Although AF has mainly been described with hyponatremia in patients with systolic HF in previous study (44, 45), one study by Timilsina et al. (46) described AF in association with the correction of chronic hypernatremia. Higher concentration of sodium is seen more often, for example, in patients treated with diuretics (47). Dehydration may be from gastrointestinal losses (gastroenteritis), renal losses (commonly due to diuretic therapy), and insufficient fluid intake, where behavior may play an important role (48). According to National Health and Nutrition Examination Survey (NHANES) survey data (49), US adults are drinking less water than is recommended. The evaluation of hydration status using body mass analysis and concentration of sodium and creatinine should be the rule in HF patient with AF. In a study of Swerdel et al. (48), the rate of dehydration in AF patients was ~9%. In this study, 18- to 80-year-old patients with AF and with comorbid dehydration had a 60% higher risk of ischemic stroke compared with AF patients without comorbid dehydration [absolute risk reduction (ARR) 1.60, 95% CI 1.28–2.00] within 10 days post-AF discharge and 34% higher risk of ischemic stroke in days 11–20 post-AF discharge (ARR 1.34, 95% CI 1.04, 1.74) (48). In a study of Groh et al. (50), the most commonly reported triggers of AF were alcohol (35%), caffeine (28%), exercise (23%), and dehydration (21%). Further studies are needed to determine the importance of various BIA parameters, including hydration status, for the occurrence of AF and prognosis in patients with HF.

The presented analysis is to our best knowledge the first analysis of HF patients to evaluate the overall impact of AF on exercise capacity and quality of life and to assess the relations between body mass compartments and AF occurrence in HF patients. This analysis pays attention to the fact that HF patients with AF are older, less thirsty, and more often treated with diuretics, and as a result, they are more dehydrated, with lower percentage of H_2_O-TBW, higher creatinine concentration, and higher sodium level. We demonstrated that dehydration also played an important role in HF patients with AF, and hydration status should be considered in overall HF patient care.

## Conclusion

The presented data provide an analysis of HF patients by distinguishing the dominant rhythm in ECG. The results indicate the need for improvement in the field of HF care in the subpopulation of AF patients. Among the patients with HF, those with AF had significantly lower exercise tolerance, reflected in lower VO_2_max, VO_2_max/kg, VO_2_%, and VO_2_/HR. They also had impaired ventilatory efficiency (higher VE/VCO_2_ slope) and lower submaximal exercise capacity (VO_2_ AT). HF patients with AF were paradoxically often more dehydrated, with lower TBW%, higher creatinine concentration, and higher sodium level. They also had a significantly higher level of biomarkers associated with inflammation (CRP and hs-CRP) and myocyte stress and injury (NT-proBNP and hs-cTnT) and RV systolic dysfunction in echocardiography than non-AF individuals. In multifactorial analysis, higher BMI (OR 1.23 per unit increase), greater LAVI (OR 1.07 per unit increase), lower TAPSE (OR 0.74 per unit increase), and lower TBW% in body mass analysis (OR 0.90 per unit increase) were independently related to AF in patients with HF.

Personalized diagnostic process using the CPET, 6MWT, and BIA to examine the hemodynamic and clinical consequences of AF in individual patients may help identify those who may benefit more from a rhythm control strategy. AF has not been previously described in association with dehydration and hypernatremia. The potential association of lower H_2_O-TBW% and higher BMI with AF may indicate the need for optimal hydration levels and body composition for patients with HF.

### Study Limitations and Strengths

There are several limitations of the present study, which included a small study population without healthy controls. Of 113 patients, 23 had AF, the remaining 90 were in SR, and paired analysis was possible in 23 cases only. This was also a non-randomized study. Clinical background and sample sizes were dissimilar for AF and SR, because of the natural incidence of AF in HF population. However, the data were prospectively collected from the patients. Moreover, we conducted only ECG rhythm-related statistical analysis but did not perform analysis by EF value (HFrEF vs. HFpEF, and HFmrEF). In addition, the patient population was limited to those hospitalized due to HF in our department, which could have caused referral bias because patients referred for hospitalization are not representative of the general AF and HF population. Because the disease severity in our patients was mild or moderate, without HF and AF, the results should be carefully interpreted when applied to different populations. In addition, the study design was limited regarding the evaluation of the effect of medications. Among them, the medication of BBs should be carefully considered because it might significantly affect the behavior of HR. Finally, the present study only included stable patients who were able to undergo CPET. Therefore, the present results cannot be extrapolated to unstable AF patients or with decompensated HF. To summarize, the present results need to be treated with caution and should be reproduced in a larger HF population with AF, preferably in a prospective controlled clinical trial, to confirm its results.

Despite these limitations, the presented analysis is to our best knowledge the first analysis of HF patients to evaluate the overall impact of AF on exercise capacity and quality of life and to assess the relations between body mass compartments and AF occurrence in HF patients. We demonstrated that dehydration also played an important role in HF patients with AF and hydration status should be considered in overall HF patient care.

Moreover, the paradox of a lower VO_2_ at AT in AF patients in our study raises uncertainties about the use of AT data to define performance and prognosis of HF patients with AF, supporting the need of multiparametric evaluation of CPET in this disease.

## Data Availability Statement

The raw data supporting the conclusions of this article will be made available by the authors, without undue reservation.

## Ethics Statement

The studies involving human participants were reviewed and approved by Polish Mother's Memorial Hospital Research Institute (PMMHRI-BCO.16/2019). The patients/participants provided their written informed consent to participate in this study.

## Author Contributions

AC contributed to conception and design of the paper, managed the literature research, and wrote the first draft of the manuscript. AB-D contributed to conception and design of the paper, managed the literature research, and reviewed and made corrections to the manuscript. MK performed a statistical analysis of the material. MM and MB contributed to conception and design of the paper and reviewed and made corrections to the manuscript. All authors contributed to manuscript final revision, read, and approved the submitted version.

## Conflict of Interest

The authors declare that the research was conducted in the absence of any commercial or financial relationships that could be construed as a potential conflict of interest.
